# *cis*-Dichlorido[2-methyl-8-(pyridin-2-ylmeth­oxy)quinoline-κ^3^ *N*,*O*,*N*′](triphenylphosphane-κ*P*)ruthenium(II) methanol monosolvate

**DOI:** 10.1107/S1600536810047033

**Published:** 2010-11-27

**Authors:** Hui-Jun Xu, Yu Li, Qing-Yang Du

**Affiliations:** a School of Chemical Engineering, Shandong University of Technology, 255049 Zibo, Shandong, People’s Republic of China; b School of Materials Science and Engineering, Shandong University of Technology, 255049 Zibo, Shandong, People’s Republic of China

## Abstract

In the structure of the title compound, [RuCl_2_(C_16_H_14_N_2_O)(C_18_H_15_P)]·CH_3_OH, he Ru^II^ ion shows a slightly distorted octahedral coordination by two N atoms and one O atom from the 2-methyl-8-(pyridin-2-ylmeth­oxy)quinoline acting as an *N*,*O*,*N*′-tridentate ligand, two Cl atoms, and one P atom from a PPh_3_ ligand. The two Cl atoms adopt a *cis* arrangement with the PPh_3_ ligand *trans* to one Cl atom. The *N*,*O*,*N*′-tridentate ligand occupies a *mer* position in the coordination sphere.

## Related literature

For related structures, see: Al-Mandhary & Steel (2003 ▶); Deng *et al.* (2005 ▶); Xu *et al.* (2009 ▶).
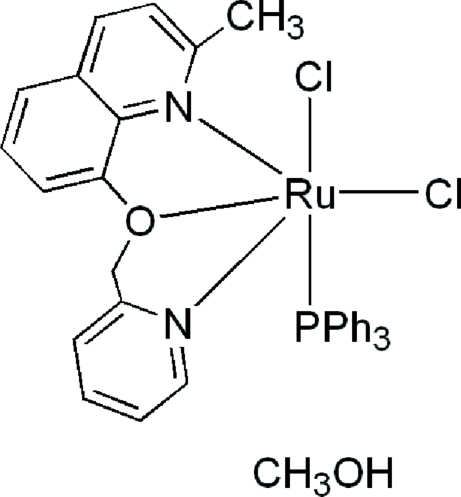


## Experimental

### 

#### Crystal data

[RuCl_2_(C_16_H_14_N_2_O)(C_18_H_15_P)]·CH_4_O
*M* *_r_* = 716.57
Orthorhombic, 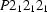

*a* = 8.868 (2) Å
*b* = 11.480 (2) Å
*c* = 31.6351 (18) Å
*V* = 3220.7 (10) Å^3^
*Z* = 4
Mo *K*α radiation
μ = 0.74 mm^−1^
*T* = 291 K
0.28 × 0.24 × 0.22 mm

#### Data collection

Bruker SMART APEX CCD diffractometer
Absorption correction: multi-scan (*SADABS*; Sheldrick, 2003 ▶) *T*_min_ = 0.820, *T*_max_ = 0.855
17669 measured reflections
6304 independent reflections
5620 reflections with *I* > 2σ(*I*)
*R*_int_ = 0.030

#### Refinement

*R*[*F* ^2^ > 2σ(*F* ^2^)] = 0.049
*wR*(*F* ^2^) = 0.117
*S* = 1.10
6304 reflections
391 parameters
H-atom parameters constrained
Δρ_max_ = 0.70 e Å^−3^
Δρ_min_ = −0.91 e Å^−3^
Absolute structure: Flack (1983 ▶), 2722 Friedel pairs
Flack parameter: 0.06 (4)

### 

Data collection: *SMART* (Bruker, 2003 ▶); cell refinement: *SAINT-Plus* (Bruker, 2003 ▶); data reduction: *SAINT-Plus*; program(s) used to solve structure: *SHELXS97* (Sheldrick, 2008 ▶); program(s) used to refine structure: *SHELXL97* (Sheldrick, 2008 ▶); molecular graphics: *SHELXTL* (Sheldrick, 2008 ▶); software used to prepare material for publication: *SHELXTL*.

## Supplementary Material

Crystal structure: contains datablocks global, I. DOI: 10.1107/S1600536810047033/jh2228sup1.cif

Structure factors: contains datablocks I. DOI: 10.1107/S1600536810047033/jh2228Isup2.hkl

Additional supplementary materials:  crystallographic information; 3D view; checkCIF report

## Figures and Tables

**Fig. 1.**
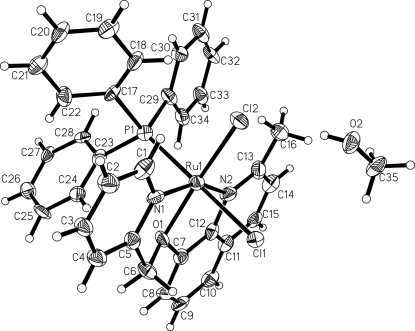
The structure of 1, with atom lables and 30% probability displacement ellipsoids.

## supplementary crystallographic information

### Comment

The N,*O*,*N*'-tridentate ligands with two pyridine-like donors and an ether donor are potentially a doubly chelating ligands in coordination chemistry. The presence of the flexible methylene and ether linkages allows the ligands to act as a folded N,*N*'-bidentate ligands or N,*O*,*N*'-tridentate ligands in meridional or facial arrangement, coordination to the metal coordinate (Al-Mandhary & Steel, 2003; Xu *et al.* 2009). Here, we report the synthesis and crystal structure of the title complex 1, [Ru(C_16_H_14_N_2_O)(PPh_3_)Cl_2_.CH_3_OH, which combines 2-methyl-8-(pyridineyl-2-methoxy)-quinoline and triphenylphosphane ligands. The molecular structure of the title compound is shown in Fig. 1. In the title complex 1, the ruthenium atom center is in a pseudooctahedral environment with the two nitrogen atoms and one oxygen atom which from 2-methyl-8-(pyridineyl-2-methoxy)-quinoline acts as a *mer* N,*O*,*N*'-tridentate ligand, two *cis* chlorine atoms and one phosphorus atom from PPh_3_ ligand *trans* to one chloride. The N—Ru—N angle is 158.25 (17)°. The N1—Ru—O1 and N2—Ru—O1 angles are 79.47 (17)° and 79.66 (16)°, respectively. The coordination between N,*O*,*N*'-tridentate ligand and the Ru^II^ yields two five-membered rings, RuN1C6C5O1 and RuO1C7C12N2. The Ru—Cl1 distance [2.4905 (14) Å], which is *trans* to the PPh_3_ ligand, is longer than the Ru—Cl2 distance [2.4104 (14) Å], similar differences are in agreement with reported value (Deng *et al.* 2005; Xu *et al.* 2009). The Ru—*N*(pyridine) [2.067 (5) Å], Ru—*N*(quinoline) [2.163 (5) Å], Ru—O [2.060 (3) Å] and Ru—P [2.2931 (15) Å] are similar to reported value (Deng *et al.* 2005; Xu *et al.* 2009).

### Experimental

The synthesis of the title compound 1 was carried out as follows. 2-methyl-8-(pyridineyl-2-methoxy)-quinoline (0.286 g, 0.55 mmol) was added to a solution of RuCl_2_(PPh_3_)_3_ (0.491 g, 0.50 mmol) in CH_3_OH (25 ml). This mixture was refluxed for 8 h and then evaporated to dryness. The orange solid residue was dissolved in CH_2_Cl_2_ (*ca* 3 ml) and the resulting solution was transferred to a silica gel chromatography column. Elution with CH_2_Cl_2_/CH_3_OH (40:1) gave a yellow-orange band, from which complex 1 was obtained after solvent removal. Crystal of 1 suitable for X-ary structure determination was grown from CH_3_OH solution of the complex layered with ethyl ether.

### Refinement

H atoms on C atoms were placed in idealized positions (C—H = 0.93—0.97 Å) and refined as riding atoms, with the *U*_iso_(H) = 1.2 or 1.5*U*_eq_(C). H atom attached to O atom are located in a difference Fourier map and refined as riding in their 'as found' positions with the *U*_iso_(H) = 1.5*U*_eq_(O).

### Crystal data

**Table:**

[RuCl_2_(C_16_H_14_N_2_O)(C_18_H_15_P)]·CH_4_O	*F*(000) = 1464
*M**_r_* = 716.57	*D*_x_ = 1.478 Mg m^−^^3^
Orthorhombic, *P*2_1_2_1_2_1_	Mo *K*α radiation, λ = 0.71073 Å
Hall symbol: P 2ac 2ab	Cell parameters from 592 reflections
*a* = 8.868 (2) Å	θ = 2.4–13.2°
*b* = 11.480 (2) Å	µ = 0.74 mm^−^^1^
*c* = 31.6351 (18) Å	*T* = 291 K
*V* = 3220.7 (10) Å^3^	Block, orange
*Z* = 4	0.28 × 0.24 × 0.22 mm

### Data collection

**Table:**

Bruker SMART APEX CCD diffractometer	6304 independent reflections
Radiation source: sealed tube	5620 reflections with *I* > 2σ(*I*)
graphite	*R*_int_ = 0.030
phi and ω scans	θ_max_ = 26.0°, θ_min_ = 1.9°
Absorption correction: multi-scan (*SADABS*; Sheldrick, 2003)	*h* = −10→9
*T*_min_ = 0.820, *T*_max_ = 0.855	*k* = −14→10
17669 measured reflections	*l* = −38→38

### Refinement

**Table:**

Refinement on *F*^2^	Secondary atom site location: difference Fourier map
Least-squares matrix: full	Hydrogen site location: inferred from neighbouring sites
*R*[*F*^2^ > 2σ(*F*^2^)] = 0.049	H-atom parameters constrained
*wR*(*F*^2^) = 0.117	*w* = 1/[σ^2^(*F*_o_^2^) + (0.06*P*)^2^ + 1.99*P*] where *P* = (*F*_o_^2^ + 2*F*_c_^2^)/3
*S* = 1.10	(Δ/σ)_max_ < 0.001
6304 reflections	Δρ_max_ = 0.70 e Å^−^^3^
391 parameters	Δρ_min_ = −0.91 e Å^−^^3^
0 restraints	Absolute structure: Flack (1983), 2722 Friedel pairs
Primary atom site location: structure-invariant direct methods	Flack parameter: 0.06 (4)

### Special details

**Table:**

Geometry. All e.s.d.'s (except the e.s.d. in the dihedral angle between two l.s. planes) are estimated using the full covariance matrix. The cell e.s.d.'s are taken into account individually in the estimation of e.s.d.'s in distances, angles and torsion angles; correlations between e.s.d.'s in cell parameters are only used when they are defined by crystal symmetry. An approximate (isotropic) treatment of cell e.s.d.'s is used for estimating e.s.d.'s involving l.s. planes.
Refinement. Refinement of *F*^2^ against ALL reflections. The weighted *R*-factor *wR* and goodness of fit *S* are based on *F*^2^, conventional *R*-factors *R* are based on *F*, with *F* set to zero for negative *F*^2^. The threshold expression of *F*^2^ > σ(*F*^2^) is used only for calculating *R*-factors(gt) *etc*. and is not relevant to the choice of reflections for refinement. *R*-factors based on *F*^2^ are statistically about twice as large as those based on *F*, and *R*- factors based on ALL data will be even larger.

### Fractional atomic coordinates and isotropic or equivalent isotropic displacement parameters (Å^2^)

**Table:**

	*x*	*y*	*z*	*U*_iso_*/*U*_eq_	
C1	0.1967 (7)	0.4572 (6)	0.6458 (2)	0.0547 (15)	
H1	0.1733	0.4993	0.6216	0.066*	
C2	0.0977 (8)	0.3754 (6)	0.6601 (2)	0.0547 (16)	
H2	0.0091	0.3628	0.6451	0.066*	
C3	0.1231 (7)	0.3129 (5)	0.69486 (18)	0.0481 (14)	
H3	0.0548	0.2561	0.7033	0.058*	
C4	0.2514 (7)	0.3333 (5)	0.71807 (18)	0.0482 (14)	
H4	0.2707	0.2934	0.7431	0.058*	
C5	0.3511 (6)	0.4166 (5)	0.70246 (16)	0.0396 (11)	
C6	0.4939 (7)	0.4460 (5)	0.72707 (17)	0.0455 (12)	
H6A	0.4734	0.5064	0.7477	0.055*	
H6B	0.5309	0.3776	0.7418	0.055*	
C7	0.7414 (7)	0.5321 (5)	0.71092 (17)	0.0406 (12)	
C8	0.8219 (7)	0.4838 (5)	0.74429 (15)	0.0424 (13)	
H8	0.7791	0.4250	0.7606	0.051*	
C9	0.9665 (7)	0.5233 (5)	0.75332 (18)	0.0512 (17)	
H9	1.0204	0.4910	0.7757	0.061*	
C10	1.0306 (6)	0.6112 (5)	0.72899 (18)	0.0499 (15)	
H10	1.1273	0.6376	0.7350	0.060*	
C11	0.9500 (6)	0.6594 (5)	0.69561 (19)	0.0416 (13)	
C12	0.8054 (6)	0.6199 (5)	0.68658 (18)	0.0409 (12)	
C13	0.7889 (7)	0.7560 (5)	0.6289 (2)	0.0498 (15)	
C14	0.9335 (6)	0.7956 (5)	0.63790 (18)	0.0481 (14)	
H14	0.9764	0.8543	0.6216	0.058*	
C15	1.0141 (8)	0.7473 (4)	0.67127 (15)	0.0461 (13)	
H15	1.1108	0.7737	0.6773	0.055*	
C16	0.6974 (8)	0.8152 (6)	0.59660 (19)	0.0556 (16)	
H16A	0.6168	0.7648	0.5878	0.083*	
H16B	0.7595	0.8337	0.5727	0.083*	
H16C	0.6561	0.8855	0.6082	0.083*	
C17	0.4278 (6)	0.3833 (5)	0.56457 (16)	0.0389 (12)	
C18	0.3214 (7)	0.4467 (5)	0.54310 (19)	0.0492 (15)	
H18	0.3255	0.5276	0.5434	0.059*	
C19	0.2062 (7)	0.3896 (6)	0.52066 (19)	0.0520 (16)	
H19	0.1352	0.4319	0.5054	0.062*	
C20	0.2012 (7)	0.2744 (5)	0.5218 (2)	0.0510 (15)	
H20	0.1250	0.2367	0.5069	0.061*	
C21	0.3002 (7)	0.2094 (6)	0.54320 (19)	0.0514 (15)	
H21	0.2917	0.1287	0.5429	0.062*	
C22	0.4163 (8)	0.2625 (5)	0.5660 (2)	0.0526 (15)	
H22	0.4840	0.2183	0.5818	0.063*	
C23	0.6972 (6)	0.3305 (5)	0.61146 (17)	0.0397 (12)	
C24	0.6582 (7)	0.2702 (5)	0.64730 (19)	0.0469 (13)	
H24	0.5781	0.2966	0.6637	0.056*	
C25	0.7358 (8)	0.1700 (5)	0.65976 (17)	0.0490 (15)	
H25	0.7136	0.1346	0.6855	0.059*	
C26	0.8444 (8)	0.1244 (5)	0.63414 (19)	0.0529 (16)	
H26	0.8935	0.0559	0.6416	0.063*	
C27	0.8805 (6)	0.1802 (4)	0.59724 (17)	0.0401 (12)	
H27	0.9554	0.1497	0.5799	0.048*	
C28	0.8063 (7)	0.2826 (5)	0.58537 (16)	0.0407 (12)	
H28	0.8302	0.3186	0.5599	0.049*	
C29	0.6986 (7)	0.5251 (5)	0.55537 (18)	0.0456 (14)	
C30	0.6518 (7)	0.5601 (5)	0.51666 (17)	0.0462 (14)	
H30	0.5546	0.5422	0.5077	0.055*	
C31	0.7457 (7)	0.6215 (5)	0.4905 (2)	0.0510 (16)	
H31	0.7118	0.6441	0.4639	0.061*	
C32	0.8927 (7)	0.6507 (5)	0.50324 (19)	0.0485 (15)	
H32	0.9544	0.6944	0.4855	0.058*	
C33	0.9447 (7)	0.6144 (5)	0.54189 (19)	0.0499 (15)	
H33	1.0441	0.6272	0.5500	0.060*	
C34	0.8443 (7)	0.5582 (5)	0.56832 (18)	0.0446 (14)	
H34	0.8742	0.5415	0.5958	0.054*	
C35	0.3573 (7)	1.0239 (6)	0.6490 (2)	0.0559 (16)	
H35A	0.3218	1.1028	0.6508	0.084*	
H35B	0.4463	1.0214	0.6317	0.084*	
H35C	0.3806	0.9956	0.6768	0.084*	
Cl1	0.43030 (16)	0.71863 (12)	0.70588 (4)	0.0412 (3)	
Cl2	0.35481 (17)	0.69468 (12)	0.60054 (5)	0.0474 (3)	
N1	0.3286 (5)	0.4787 (4)	0.66606 (13)	0.0385 (10)	
N2	0.7249 (5)	0.6682 (4)	0.65321 (14)	0.0433 (11)	
O1	0.6057 (4)	0.4866 (3)	0.69627 (11)	0.0388 (8)	
O2	0.2468 (5)	0.9549 (4)	0.63110 (15)	0.0588 (12)	
H2A	0.2852	0.8955	0.6216	0.071*	
P1	0.57943 (18)	0.45692 (12)	0.59589 (4)	0.0401 (3)	
Ru1	0.50826 (5)	0.58272 (3)	0.648543 (12)	0.03625 (11)	

### Atomic displacement parameters (Å^2^)

**Table:**

	*U*^11^	*U*^22^	*U*^33^	*U*^12^	*U*^13^	*U*^23^
C1	0.050 (3)	0.057 (4)	0.057 (4)	−0.008 (3)	−0.002 (3)	−0.018 (3)
C2	0.056 (4)	0.058 (4)	0.051 (4)	−0.014 (3)	−0.004 (3)	0.008 (3)
C3	0.054 (4)	0.044 (3)	0.047 (3)	−0.016 (3)	−0.014 (3)	0.011 (3)
C4	0.051 (3)	0.050 (3)	0.043 (3)	−0.005 (3)	−0.011 (3)	0.008 (3)
C5	0.041 (3)	0.035 (3)	0.044 (3)	0.004 (3)	0.004 (2)	0.001 (2)
C6	0.039 (3)	0.049 (3)	0.049 (3)	0.012 (3)	−0.001 (3)	0.018 (2)
C7	0.042 (3)	0.036 (3)	0.044 (3)	−0.002 (2)	−0.012 (2)	−0.004 (2)
C8	0.049 (3)	0.051 (3)	0.027 (3)	−0.012 (3)	−0.006 (2)	0.006 (2)
C9	0.057 (5)	0.058 (4)	0.038 (3)	0.015 (3)	−0.019 (3)	−0.021 (3)
C10	0.038 (3)	0.059 (4)	0.052 (3)	0.005 (3)	−0.015 (3)	−0.016 (3)
C11	0.030 (3)	0.043 (3)	0.051 (3)	−0.006 (2)	0.001 (2)	−0.009 (2)
C12	0.037 (3)	0.034 (3)	0.052 (3)	0.000 (2)	−0.006 (2)	−0.012 (2)
C13	0.057 (4)	0.036 (3)	0.056 (3)	−0.002 (3)	0.002 (3)	−0.001 (3)
C14	0.033 (3)	0.056 (3)	0.055 (3)	−0.005 (3)	0.011 (3)	−0.012 (3)
C15	0.053 (3)	0.044 (3)	0.041 (3)	0.004 (3)	0.019 (3)	−0.015 (2)
C16	0.069 (4)	0.051 (4)	0.047 (3)	−0.009 (3)	0.009 (3)	0.011 (3)
C17	0.043 (3)	0.040 (3)	0.034 (3)	0.005 (2)	0.004 (2)	−0.021 (2)
C18	0.051 (3)	0.039 (3)	0.058 (4)	−0.001 (3)	−0.018 (3)	−0.019 (3)
C19	0.043 (3)	0.059 (4)	0.054 (4)	0.015 (3)	−0.011 (3)	−0.019 (3)
C20	0.045 (3)	0.044 (3)	0.063 (4)	−0.003 (3)	−0.013 (3)	−0.022 (3)
C21	0.043 (3)	0.057 (4)	0.054 (3)	−0.011 (3)	−0.011 (3)	−0.014 (3)
C22	0.056 (4)	0.037 (3)	0.065 (4)	−0.015 (3)	−0.009 (3)	0.005 (3)
C23	0.037 (3)	0.035 (3)	0.047 (3)	−0.008 (2)	−0.009 (2)	−0.008 (2)
C24	0.057 (3)	0.039 (3)	0.044 (3)	0.001 (2)	−0.021 (3)	0.000 (3)
C25	0.067 (4)	0.045 (3)	0.035 (3)	0.005 (3)	0.001 (3)	0.024 (2)
C26	0.069 (4)	0.037 (3)	0.053 (3)	0.022 (3)	−0.009 (3)	0.003 (3)
C27	0.041 (3)	0.031 (3)	0.048 (3)	0.013 (2)	−0.012 (2)	−0.016 (2)
C28	0.051 (3)	0.046 (3)	0.025 (2)	0.000 (3)	−0.012 (2)	−0.004 (2)
C29	0.053 (3)	0.040 (3)	0.044 (3)	0.015 (3)	0.009 (3)	0.013 (3)
C30	0.055 (4)	0.045 (3)	0.039 (3)	0.004 (3)	0.001 (3)	0.003 (2)
C31	0.049 (3)	0.046 (3)	0.058 (4)	0.025 (3)	0.019 (3)	0.017 (3)
C32	0.048 (4)	0.047 (3)	0.051 (3)	0.016 (3)	0.013 (3)	0.026 (3)
C33	0.056 (4)	0.041 (3)	0.054 (3)	0.015 (2)	0.014 (3)	0.006 (3)
C34	0.055 (3)	0.034 (3)	0.044 (3)	0.007 (2)	0.020 (3)	0.006 (2)
C35	0.064 (4)	0.057 (4)	0.046 (3)	−0.013 (3)	0.023 (3)	−0.020 (3)
Cl1	0.0449 (7)	0.0410 (7)	0.0376 (6)	−0.0005 (6)	−0.0036 (6)	−0.0007 (5)
Cl2	0.0492 (8)	0.0461 (8)	0.0470 (7)	−0.0014 (6)	−0.0080 (6)	0.0143 (6)
N1	0.047 (3)	0.043 (3)	0.0254 (19)	−0.003 (2)	−0.0019 (19)	−0.0066 (18)
N2	0.047 (3)	0.048 (3)	0.035 (2)	−0.006 (2)	−0.007 (2)	−0.006 (2)
O1	0.045 (2)	0.0283 (18)	0.043 (2)	−0.0104 (15)	−0.0085 (17)	0.0118 (16)
O2	0.059 (3)	0.048 (3)	0.069 (3)	0.022 (2)	−0.015 (2)	−0.021 (2)
P1	0.0446 (8)	0.0387 (7)	0.0369 (7)	0.0004 (6)	−0.0016 (6)	−0.0002 (6)
Ru1	0.0366 (2)	0.03698 (19)	0.03518 (19)	−0.0014 (2)	−0.0037 (2)	0.00081 (16)

### Geometric parameters (Å, °)

**Table:**

C1—N1	1.356 (8)	C19—H19	0.9300
C1—C2	1.362 (9)	C20—C21	1.337 (9)
C1—H1	0.9300	C20—H20	0.9300
C2—C3	1.332 (8)	C21—C22	1.398 (8)
C2—H2	0.9300	C21—H21	0.9300
C3—C4	1.374 (8)	C22—H22	0.9300
C3—H3	0.9300	C23—C24	1.373 (8)
C4—C5	1.393 (8)	C23—C28	1.385 (8)
C4—H4	0.9300	C23—P1	1.855 (6)
C5—N1	1.369 (7)	C24—C25	1.397 (8)
C5—C6	1.524 (8)	C24—H24	0.9300
C6—O1	1.466 (7)	C25—C26	1.363 (9)
C6—H6A	0.9700	C25—H25	0.9300
C6—H6B	0.9700	C26—C27	1.370 (8)
C7—C8	1.390 (8)	C26—H26	0.9300
C7—C12	1.390 (8)	C27—C28	1.398 (7)
C7—O1	1.391 (7)	C27—H27	0.9300
C8—C9	1.390 (9)	C28—H28	0.9300
C8—H8	0.9300	C29—C30	1.354 (8)
C9—C10	1.390 (9)	C29—C34	1.407 (9)
C9—H9	0.9300	C29—P1	1.837 (6)
C10—C11	1.390 (8)	C30—C31	1.369 (8)
C10—H10	0.9300	C30—H30	0.9300
C11—C12	1.390 (8)	C31—C32	1.404 (9)
C11—C15	1.390 (8)	C31—H31	0.9300
C12—N2	1.390 (7)	C32—C33	1.372 (8)
C13—N2	1.390 (8)	C32—H32	0.9300
C13—C14	1.390 (9)	C33—C34	1.382 (8)
C13—C16	1.470 (9)	C33—H33	0.9300
C14—C15	1.390 (9)	C34—H34	0.9300
C14—H14	0.9300	C35—O2	1.381 (7)
C15—H15	0.9300	C35—H35A	0.9600
C16—H16A	0.9600	C35—H35B	0.9600
C16—H16B	0.9600	C35—H35C	0.9600
C16—H16C	0.9600	Cl1—Ru1	2.4905 (14)
C17—C18	1.371 (8)	Cl2—Ru1	2.4104 (14)
C17—C22	1.392 (8)	N1—Ru1	2.067 (5)
C17—P1	1.872 (6)	N2—Ru1	2.162 (5)
C18—C19	1.406 (8)	O1—Ru1	2.060 (3)
C18—H18	0.9300	O2—H2A	0.8200
C19—C20	1.325 (9)	P1—Ru1	2.2931 (15)
			
N1—C1—C2	121.7 (7)	C24—C23—P1	118.2 (4)
N1—C1—H1	119.2	C28—C23—P1	123.1 (4)
C2—C1—H1	119.2	C23—C24—C25	121.6 (6)
C3—C2—C1	122.4 (7)	C23—C24—H24	119.2
C3—C2—H2	118.8	C25—C24—H24	119.2
C1—C2—H2	118.8	C26—C25—C24	119.8 (5)
C2—C3—C4	119.3 (6)	C26—C25—H25	120.1
C2—C3—H3	120.4	C24—C25—H25	120.1
C4—C3—H3	120.4	C25—C26—C27	119.5 (5)
C3—C4—C5	117.0 (5)	C25—C26—H26	120.2
C3—C4—H4	121.5	C27—C26—H26	120.2
C5—C4—H4	121.5	C26—C27—C28	120.8 (5)
N1—C5—C4	124.3 (5)	C26—C27—H27	119.6
N1—C5—C6	115.8 (5)	C28—C27—H27	119.6
C4—C5—C6	119.9 (5)	C23—C28—C27	120.2 (5)
O1—C6—C5	107.0 (4)	C23—C28—H28	119.9
O1—C6—H6A	110.3	C27—C28—H28	119.9
C5—C6—H6A	110.3	C30—C29—C34	117.7 (6)
O1—C6—H6B	110.3	C30—C29—P1	125.5 (5)
C5—C6—H6B	110.3	C34—C29—P1	116.1 (4)
H6A—C6—H6B	108.6	C29—C30—C31	120.8 (6)
C8—C7—C12	120.0 (5)	C29—C30—H30	119.6
C8—C7—O1	123.2 (5)	C31—C30—H30	119.6
C12—C7—O1	116.2 (5)	C30—C31—C32	120.9 (6)
C9—C8—C7	120.0 (5)	C30—C31—H31	119.5
C9—C8—H8	120.0	C32—C31—H31	119.5
C7—C8—H8	120.0	C33—C32—C31	119.7 (6)
C8—C9—C10	120.0 (5)	C33—C32—H32	120.1
C8—C9—H9	120.0	C31—C32—H32	120.1
C10—C9—H9	120.0	C32—C33—C34	117.7 (6)
C11—C10—C9	120.0 (5)	C32—C33—H33	121.2
C11—C10—H10	120.0	C34—C33—H33	121.2
C9—C10—H10	120.0	C33—C34—C29	122.8 (6)
C12—C11—C10	120.0 (5)	C33—C34—H34	118.6
C12—C11—C15	120.0 (6)	C29—C34—H34	118.6
C10—C11—C15	120.0 (5)	O2—C35—H35A	109.5
C11—C12—N2	120.0 (5)	O2—C35—H35B	109.5
C11—C12—C7	120.0 (5)	H35A—C35—H35B	109.5
N2—C12—C7	120.0 (5)	O2—C35—H35C	109.5
N2—C13—C14	120.0 (6)	H35A—C35—H35C	109.5
N2—C13—C16	119.6 (6)	H35B—C35—H35C	109.5
C14—C13—C16	120.1 (6)	C1—N1—C5	115.3 (5)
C13—C14—C15	120.0 (6)	C1—N1—Ru1	130.1 (4)
C13—C14—H14	120.0	C5—N1—Ru1	114.5 (4)
C15—C14—H14	120.0	C13—N2—C12	120.0 (5)
C14—C15—C11	120.0 (6)	C13—N2—Ru1	130.9 (4)
C14—C15—H15	120.0	C12—N2—Ru1	109.1 (4)
C11—C15—H15	120.0	C7—O1—C6	118.9 (4)
C13—C16—H16A	109.5	C7—O1—Ru1	113.9 (3)
C13—C16—H16B	109.5	C6—O1—Ru1	111.9 (3)
H16A—C16—H16B	109.5	C35—O2—H2A	109.5
C13—C16—H16C	109.5	C29—P1—C23	101.2 (3)
H16A—C16—H16C	109.5	C29—P1—C17	103.7 (3)
H16B—C16—H16C	109.5	C23—P1—C17	101.1 (2)
C18—C17—C22	119.7 (5)	C29—P1—Ru1	113.4 (2)
C18—C17—P1	121.1 (4)	C23—P1—Ru1	117.07 (18)
C22—C17—P1	119.0 (5)	C17—P1—Ru1	118.10 (17)
C17—C18—C19	120.2 (5)	O1—Ru1—N1	79.47 (17)
C17—C18—H18	119.9	O1—Ru1—N2	79.66 (16)
C19—C18—H18	119.9	N1—Ru1—N2	158.25 (17)
C20—C19—C18	118.5 (6)	O1—Ru1—P1	94.55 (11)
C20—C19—H19	120.8	N1—Ru1—P1	92.46 (12)
C18—C19—H19	120.8	N2—Ru1—P1	95.22 (13)
C19—C20—C21	123.3 (6)	O1—Ru1—Cl2	169.80 (11)
C19—C20—H20	118.4	N1—Ru1—Cl2	92.40 (14)
C21—C20—H20	118.4	N2—Ru1—Cl2	107.61 (13)
C20—C21—C22	120.2 (6)	P1—Ru1—Cl2	91.93 (6)
C20—C21—H21	119.9	O1—Ru1—Cl1	85.32 (11)
C22—C21—H21	119.9	N1—Ru1—Cl1	87.29 (12)
C17—C22—C21	118.1 (6)	N2—Ru1—Cl1	84.98 (13)
C17—C22—H22	121.0	P1—Ru1—Cl1	179.74 (6)
C21—C22—H22	121.0	Cl2—Ru1—Cl1	88.17 (5)
C24—C23—C28	117.9 (5)		
